# Assessment of the Role of Renal Organic Anion Transporters in Drug-Induced Nephrotoxicity

**DOI:** 10.3390/toxins2082055

**Published:** 2010-08-09

**Authors:** Yohannes Hagos, Natascha A. Wolff

**Affiliations:** 1 Abteilung Vegetative Physiologie & Pathophysiologie, Zentrum Physiologie & Pathophysiologie, Georg-August-Universität Göttingen, Humboldtallee 23, 37073 Göttingen, Germany; Email: Hagos@physiol.med.uni-goettingen.de; 2 Institut für Physiologie & Pathophysiologie, Zentrum für biomedizinische Ausbildung und Forschung (ZBAF), Fakultät für Medizin, Universität Witten/Herdecke, Stockumer Str. 12, D-58453 Witten, Germany

**Keywords:** drug-induced nephrotoxicity, tubular cell toxicity, nephrolithiasis, OAT1, OAT2, OAT3, OAT4

## Abstract

In the present review we have attempted to assess the involvement of the organic anion transporters OAT1, OAT2, OAT3, and OAT4, belonging to the SLC22 family of polyspecific carriers, in drug-induced renal damage in humans. We have focused on drugs with widely recognized nephrotoxic potential, which have previously been reported to interact with OAT family members, and whose underlying pathogenic mechanism suggests the participation of tubular transport. Thus, only compounds generally believed to cause kidney injury either by means of direct tubular toxicity or crystal nephropathy have been considered. For each drug, or class of agents, the evidence for actual transport mediated by individual OATs under *in vivo* conditions is discussed. We have then examined their role in the context of other carriers present in the renal proximal tubule sharing certain substrates with OATs, as these are critical determinants of the overall contribution of OAT-dependent transport to intracellular accumulation and transepithelial drug secretion, and thus the impact it may have in drug-induced nephrotoxicity.

## 1. Specific Renal Vulnerability

A variety of pathogenic mechanisms play a role in drug-induced nephrotoxicity, including hemodynamic changes, glomerular disease, interstitial nephritis, direct cytotoxicity which may result in tubular cell death, and intratubular precipitation of drugs leading to obstructive nephropathy [1,2,3]. High delivery of blood-borne substances, as well as concentration of xenobiotics entering the tubular lumen in the course of their tubular passage, in particular under conditions of dehydration, contribute to the particular vulnerability of the kidneys to injury by clinically relevant drugs, as well as environmental toxins. A large number of secretory transporters in the renal proximal tubule, many of them polyspecific, contribute not only to high intratubular solute concentrations, but also to exposure of the tubular epithelium to high intracellular levels of potential cytotoxins. The latter may be further aggravated for compounds which are additionally reabsorbed from the tubular fluid. High metabolic activity may in part account for the sensitivity of proximal tubule cells to damage by such agents known to interfere with mitochondrial substrate utilization or to decrease cellular antioxidant capacity.

Several organic anion transporters (OATs) of the SLC22 family of solute carriers, many of which accept a markedly broad spectrum of substrates, are present on either side of the renal proximal tubular epithelium. There, OAT-mediated transport can contribute to two types of renal injury: (i) direct cellular toxicity by allowing access of damaging agents such as antiviral drugs to the cytosol, as well as (ii) crystal nephropathy by mediating transepithelial secretion of compounds with low solubility in urine that tend to precipitate upon urinary concentration, such as methotrexate or acyclovir [4]. In the present review, we have focused on selected OAT drug substrates, for which clinically relevant tubulotoxicity and/or nephrolithiasis have been reported, and have attempted to assess the contribution of individual OATs to these processes in the context of additional proximal tubular transport pathways for the respective compounds. We have thus limited ourselves to those OAT family members known to interact with the nephrotoxic pharmaceuticals discussed. For a more comprehensive overview of the pharmacological, as well as physiological, relevance of SLC22 organic anion transporters, the interested reader is referred to the excellent extensive reviews by Sweet [5], Rizwan and Burckhardt [6], and van Wert *et al.* [7]. 

## 2. Involvement of OATs in Renal Proximal Tubular Solute Uptake and Transepithelial Secretion

In humans, OAT1 (SLC22A6), OAT2 (SLC22A7) and OAT3 (SLC22A8) are present in the basolateral membrane of renal proximal tubule cells [8,9,10,11]. OAT1 and OAT3 operate as exchangers for intracellular dicarboxylate [12,13,14], originating mainly from basolateral import via Na^+^-driven uptake mediated by NaDC-3 as well as cellular metabolism, while uptake of dicarboxylate from the tubular lumen—at least in the rabbit—appears to play only a minor role in the energetics of OAT1- and OAT3-mediated transport [15]. In rat renal cortical tissue, the concentration of α-ketoglutarate (α-KG) as the most likely endogenous dicarboxylate exchanging for extracellular organic anions via OAT1 and OAT3 [16] has been estimated to be in the range of 265 µM [17], and the affinity of overall basolateral transport in rat renal basolateral membrane vesicles, mediated by both Oat1 and Oat3 is in the range of 150 µM [17]. The affinity of human OAT1 for the non-metabolizable analogue glutarate is high, with a K_m_ of about 11 µM [18]. Thus, both OAT1 and OAT3 are likely to mediate influx of drugs into renal proximal tubular cells under *in vivo* conditions, even if not all of the cellular dicarboxylate may be readily available for exchange [17]. The mRNA expression level of hOAT3 is almost 3-fold higher than that of hOAT1 [8]. If this difference were to translate into corresponding differences at the protein level, hOAT3 would be expected to predominate in the basolateral uptake of substrates for which hOAT1 and hOAT3 show similar affinities and turnover rates, and may still contribute significantly to the influx of compounds preferentially transported by hOAT1.

**Figure 1:**
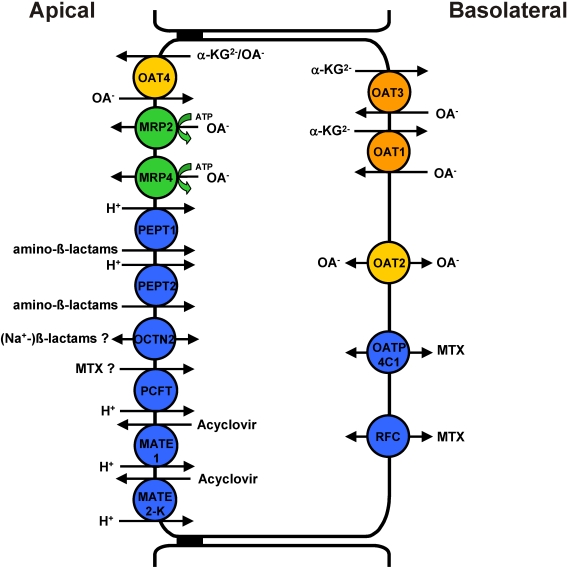
Human transporters involved in proximal tubular handling of nephrotoxic drugs discussed in the present review. OAT family members are shown in orange and yellow, ATP-driven pumps in green, all other carriers in blue. OA^−^: organic anion; α-KG: α-ketoglutarate; MTX: methotrexate; OAT: organic anion transporter; MRP: multidrug resistance-associated protein; PEPT: peptide transporter; OCTN2: novel organic cation transporter type 2; PCFT: proton-coupled folate transporter; MATE: multidrug and toxin extrusion protein; OATP: organic anion-transporting polypeptide; RFC: reduced folate carrier.

While hOAT2 has also been reported to transport α-KG and glutarate [19,20], hOAT2-mediated organic anion exchange for dicarboxylates has never been convincingly demonstrated. Thus, hOAT2 may be more likely to function as an efflux mechanism for anionic solutes reabsorbed from the tubular lumen, but could also constitute a back-leak pathway into the circulation for substrates shared with OAT1 and/or OAT3, such as methotrexate or, possibly, cephalosporin antibiotics. OAT2 mRNA expression in the human renal cortex, however, amounts to less than 15% of that determined for hOAT1 [8], and its overall contribution to proximal tubular organic anion handling may therefore be limited.

OAT4 (SLC22A11), localized at the apical side of the proximal tubule cells, is known to function as an exchanger for dicarboxylates, albeit with a low glutarate affinity (IC_50_ value 1.25 mM, determined at a substrate concentration of 50 nM)[21]. Thus, depending in part on its affinity relative to OAT1 and OAT3 for a given compound, OAT4 may either contribute to its cellular accumulation by uptake from the tubular fluid, or mediate luminal efflux in the process of transepithelial secretion. However, hOAT4 has been shown to be asymmetric in function at least for some substrates, promoting efflux but not uptake of glutarate and PAH [22]. While this would challenge the importance of OAT4 in mediating direct tubular cytotoxicity, it is unclear at present whether outwardly directed transport also predominates for other substrates. Nevertheless, mRNA expression of OAT4 in human kidney cortex is even lower than that of OAT2 [8], suggesting a minor involvement in drug-induced nephrotoxicity also for this carrier.

All four OATs mentionened above are sensitive to inhibition by the classical organic anion transport inhibitor probenecid, albeit with different affinities. While K_i_ or IC_50_ values in the range of 4.3–12.5 µM and 4–9 µM have been determined for hOAT1 and hOAT3, respectively, the affinity was determined to be somewhat lower for hOAT4 (44–68 µM), and even less for hOAT2 (766 µM)[6,23]. Probenecid has been frequently used to verify a secretory component in the renal elimination of a certain anionic drug (see below). However, it has to be emphasized that MRP and possibly also OATP family members present in the renal tubule epithelium are likewise probenecid-sensitive [24,25].

## 3. Direct Proximal Tubular Toxicity of OAT Drug Substrates

Several drugs as well as some components of Chinese herbal medications reported to be directly toxic to proximal tubule cells, including several antivirals, ß-lactam antibiotics, or aristolochic acid, are well-known substrates of OATs, the most extensively characterized basolateral u`ptake carriers OAT1 and/or OAT3 in particular. Yet, cytotoxicity of a certain agent *in vivo* also critically depends on the presence and activity of additional transport pathways, most notably those mediating apical efflux.

### 3.1. ß-Lactam antibiotics

Among the ß-lactam antibiotics, many synthetic compounds of the cephalosporin and even more so of the penem group, developed for a broader antimicrobial spectrum and bactericidal potency, have turned out to be highly nephrotoxic, in some instances to an extent prohibiting their clinical use. ß-Lactam-induced tubular necrosis has been attributed mainly to impaired import and oxidation of monocarboxylate substrates by mitochondria, as well as to cellular antioxidant depletion and lipid peroxidation [26,27]. Most cephalosporins are excreted to a significant fraction or even predominantly via the kidneys in humans [28,29]. Renal elimination of many cephalosporins, including cefaclor, cezolin and cephalothin, was significantly attenuated by coadministration of probenecid, indicating a contribution of tubular secretion via the organic anion pathway [30]. 

A number of cephalosporins, including cephaloridine as one of the most toxic [31], have been shown to significantly inhibit human OAT1, OAT2, OAT3, as well as OAT4 [32,33,34](Table 1). Although IC_50_ values determined in one study suggested that relative to hOAT1, heterologously expressed hOAT3 may be more sensitive to inhibition by most cephalosporins tested, data for hOAT1 and hOAT3 were not directly comparable, as they were obtained at a 250-fold higher substrate concentration for hOAT1 than for hOAT3 [34]. Conversely, according to the K_i_ values reported by Takeda *et al.*, hOAT1 actually exhibited the stronger affinity for the majority of cephalosporins assayed, including cephaloridine [32]. Yet, Ueo *et al.* demonstrated that heterologous expression of hOAT3 induces significant uptake, typically several fold above control (approx. 30-fold for cephaloridine), of all cephalosporins included in their study, whereas hOAT1-dependent uptake was low (e.g., cephaloridine) or non-significant (e.g., cefaclor or cefazolin)[34]. These data suggest a considerably more important role of hOAT3 over hOAT1 in tubular cephalosporin uptake, even if transporter-induced probenecid-inhibitable cephaloridine toxicity was found to be similar for both hOAT1 and hOAT3 in a different expression system [32]. The reason for this discrepancy is unclear, but might be related to differences in expression levels and/or the long incubation time (24 h) in the latter report. In this context, it is interesting to note that while the level of overexpression of hOAT3 was determined to be about 3-fold higher than that of hOAT1 in the HEK293 cells as used by Ueo *et al.* [34], this actually more closely reflects the situation in the human renal proximal tubule, as indicated above.

In contrast, although mouse proximal tubule cells stably transfected with hOAT2 or hOAT4, like those expressing hOAT1 and hOAT3, displayed increased sensitivity to cephaloridine toxicity, they differed it that cell viability was not significantly enhanced or only marginally affected by the simultaneous presence of probenecid, respectively [33]. While for hOAT2 this result was attributed to the transporter's low probenecid affinity, no such explanation can account for the low responsiveness of hOAT4. At present, it therefore appears unlikely that OAT4 plays a role in cephaloridine toxicity, or in its transepithelial secretion, whereas OAT2 may constitute a back-leak pathway of cephalosporins into the blood.

Many cephalosporins are well-known substrates of the proton-coupled peptide transporters hPEPT2 and hPEPT1, localized at the apical membrane of the renal proximal tubule epithelium. Of the nephrotoxic cephalosporins known as OAT substrates, the aminocephalosporin cefaclor is also transported with high affinity by PEPT2, while the affinity for PEPT1 is low, although transport has been demonstrated. On the other hand, there is at best weak interaction of either carrier with cephaloridine and cefalothin, as these lack a free amino group [35,36,37]. In contrast, OCTN2, another carrier localized at the apical membrane of tubule cells [38], has been shown to be highly sensitive to inhibition by cephaloridine, as well as by a number of other cephalosporins [37]. Yet, conflicting results have been reported as for its ability to actually translocate cephaloridine [37,39]. Interestingly, however, in mice Octn2 has recently been associated with Na-independent apical cephaloridine extrusion into the tubular lumen, rather than cellular uptake [40].

**Table 1:** Interaction of drugs known to be directly toxic to proximal tubule cells with human renal OATs and other human proximal tubular transporters. All values are in µM. T: transport demonstrated; n.t.: not transported; I: inhibition shown; n.i.: no inhibition; *: K_m_; ^●^: K_i_; ^◊^: IC_50_; *^a^*: carrier-induced toxicity of compound (CC_50_, concentration inducing half-maximal toxicity); *^b^*: no transporter-mediated toxicity; *^c^*: carrier-induced resistance; *^d^*: value determined in the absence of serum.

Substrate	OAT1	OAT2	OAT3	OAT4	Other Transporters			References
					MRP2	MRP4	Additional Carriers	
**β-Lactam Antibiotics**								
Cefaclor	n.t.; ^◊^1096		T; ^◊^120			I	PEPT2: *70.2; ^●^65	[34,36,41,42]
							PEPT1: T, ^●^4520; ^●^~11000	
Cefazolin	n.t.; ^◊^101	^●^5090	T; ^◊^117		n.t.	*81		[29,33,34,42]
Cephaloridine	T; *^a^*T;	*^a^*T;	T; *^a^*T;	*^a^*T;		n.i.	PEPT2: n.i.;	[18,32,33,34,36,37,39,42]
	^●^740;	^●^2090	^●^2460;	^●^3630			PEPT1: n.i.;	
	^◊^2470;		^◊^626				OCTN2: T; ^◊^790; n.t.	
	^◊^1250							
Cephalothin	^●^220	^●^1040	^●^40	^●^200		I	PEPT2: ^◊^7500; PEPT1: ^●^14000	[32,33,35,36,42]
Imipenem	*^b^*n.t.		*^a^*T (770)					[43]
Meropenem	T		*847					[44]
**Antivirals**								
Adefovir	*23.8; *30		*1220		n.t.	* >1 mM; *^c^*T		[12,18,45,46,47]
Cidofovir	*46; *58				n.t.	n.t.		[12,46,47,48]
Tenofovir	*33.8		*770		n.t.	* >1 mM; T		[45,46,49]
**NSAIDs**								
Acetylsalicylate	^◊^769	^◊^ >2000	^◊^717	^◊^ >2000				[50]
Ibuprofen	^◊^55.6	^◊^692	^◊^6.00;	^◊^103	^◊^930	^◊^26.3; I		[50,51,52,53]
			^●,*d*^1170					
Mefenamic acid	^◊^0.83	^◊^21.7	^◊^0.78	^◊^61.7				[50]
Phenacetin	^◊^275	^◊^1878	^◊^19.4	^◊^ >2000				[50]
Phenylbutazone			^●,*d*^34.7		^◊^605	^◊^130		[51,52]
**Aristolochic acid**	^●^0.6		^●^0.5	^●^20.6				[54]

Both MRP2 and MRP4 seem to constitute luminal efflux pathways for at least some ß-lactam antibiotics, including cefazolin (MRP4 only), in the human renal proximal tubule [29,42,55], with apparent affinities in the micromolar range determined for MRP4-mediated transport of ceftizoxime and cefazolin (Table 1). Moreover, most cephalosporins tested dose-dependently inhibited MRP4-mediated transport, with the notable exception of cephaloridine [42]. This is consistent with the finding in rabbits that cephaloridine exhibits only minimal efflux into the tubular fluid, which may explain its particularly high nephrotoxicity [26,56]. Interaction of cephaloridine with MRP2 has not been tested. Whether human NPT1 is also able to mediate Na^+^-independent transport of ß-lactam antibiotics and thus contributes to their apical secretion, as reported for its mouse orthologue [57], remains to be determined.

In contrast to cephalosporins, literature regarding the renal transport of penems in humans is scarce. Imipenem, which has been shown to cause tubular necrosis in experimental animals [58,59], is used in severe polymicrobial infections and sepsis. Imipenem is eliminated primarily via the kidneys, and probenecid has been found to slightly, but significantly retard its excretion [60,61]. Lim *et al.* recently reported that expression of hOAT3, but not hOAT1, in MDCK cells induced sensitivity to imipenem toxicity with a half-maximal effect at sub-millimolar imipenem concentrations [43]. However, imipenem is commonly administered together with cilastin, which not only inhibits its degradation by the renal dehydropeptidase I, but also prevents its nephrotoxic effect [58]. Interestingly, *in vitro*, cilastin has been found to preferentially inhibit hOAT3 (K_i_ 231 µM) over hOAT1 (K_i_ 1470 µM)[62]. Likewise, panipenem, which is similarly nephrotoxic [26], is marketed as a combination drug with betamipron [63], which has been found to largely prevent panipenem nephrotoxicity in rabbits [64]. Betamipron is a potent inhibitor of both hOAT1- and hOAT3-mediated transport (K_i_ values of 23.6 and 48.3 µM, respectively), whereas hOAT4 is somewhat less affected (K_i_ 502 µM)[11,62]. In contrast to imipenem, meropenem, reported to be only mildly nephrotoxic toxic in rabbits and monkeys [65], was found to be a substrate of both hOAT1 and hOAT3 [44].

Taken together, OAT3, possibly complemented to some extent by OAT1, currently appears to be the predominant uptake pathway for those nephrotoxic ß-lactam antibiotics not belonging to the amino-ß-lactams, as well as for several carbapenems, into human renal proximal tubule cells. In contrast, the mechanisms mediating apical efflux, albeit apparently limited for some of these drugs, are less clearly defined. While MRP2 and MRP4 interact at least with some of the moderately (or non-) toxic cephalosporins, data regarding their interaction with carbapenems are lacking, and the potential contribution of OCTN2 does not seem to be fully elucidated.

### 3.2. Antiviral drugs

The treatment of severe viral infections often requires long-term drug therapy, which unfortunately is frequently associated with severe side-effects. Clinically relevant nephrotoxic adverse effects of antiviral compounds are well documented in the literature, as reviewed in [66]. Dose-limiting direct tubular toxicity has been reported in particular for the nucleotide analogues adefovir and cidofovir, used primarily for treatment of infections with DNA viruses, such as cytomegalovirus or hepatitis B virus [67,68,69,70,71]. Adefovir and cidofovir are mainly eliminated unchanged via the kidneys by a combination of glomerular filtration and tubular secretion, with secretion contributing about 60% and 35% to the renal clearance of adefovir and cidofovir, respectively [72,73]. Adefovir-induced cellular damage has been attributed to mitochondrial injury, impaired ATP synthesis and/or interference with ATP-dependent cellular mechanisms [74]. Cidofovir is believed to be toxic due to interference with cellular lipid synthesis and/or degradation [74]. Adefovir and cidofovir have both been shown to be high affinity substrates of hOAT1 [12,48], whereas they were found to be only marginally transported by hOAT3 in some studies (adefovir [75], cidofovir [76]). At least with respect to adefovir, this finding might be explained by an affinity for hOAT3 about 50-fold lower than for hOAT1 [45]. To our knowledge there are as yet no reports on the ability of hOAT2 and hOAT4—as potential efflux routes from proximal tubular cells—to interact with nucleotide analogue antivirals.

In contrast, MRP4 is well known to be able to transport adefovir, but not cidofovir [46,47], whereas MRP2 does not seem to be involved in the apical extrusion of nucleotide analogues [46]. Interestingly, the nucleotide analogue tenofovir, used in particular for the treatment of HIV infections, has originally been considered relatively safe, when compared to adefovir and cidofovir, with no evidence for tubular toxicity observed in earlier clinical trials [77,78]. Yet, similar to adefovir and cidofovir, tenofovir, which is also negatively charged under physiological conditions, is a high affinity substrate of hOAT1, albeit a rather poor substrate for hOAT3 with a high-micromolar K_m_ value [45]. Transport efficiency via hOAT1—determined in the same expression system under identical conditions—was even almost twice that of adefovir and cidofovir [45]. Transport via MRP4 as a possible apical efflux route displayed an affinity for tenofovir as low as for adefovir [46], while again no MRP2-mediated transport was observed [46,49]. Thus, the generally lower nephrotoxicity of tenofovir relative to adefovir or cidofovir at present does not seem to be attributable to differences in tubular transport, suggesting that higher cytosolic concentrations—as could result from impaired luminal efflux (see below)—might be required for tenofovir to cause cellular injury. Indeed, tenofovir has been shown to have a relatively low toxicity toward mitochondria [79].

More recently, however, an appreciable number of cases of tenofovir-induced Fanconi-like syndromes and acute renal failure have been reported, in part under combination therapy with HIV protease inhibitors, such as lopinavir/ritonavir or atazanavir [80,81,82,83]. These renal adverse effects have alternatively been associated with MRP2 polymorphisms, such as the V417I variant [84], or attributed to an interaction of the protease inhibitor with MRP2- or MRP4-mediated export [66,85,86]. The reason for the association of tenofovir-induced proximal tubular damage with the MRP2-V417I variant is as yet unclear. Although MRP2-mediated tenofovir transport might have escaped detection if MRP2 exhibited an even lower affinity for this antiviral drug than MRP4, *in vitro* MRP2-V417I did not differ functionally from wildtype-MRP2 [87], but tenofovir was not tested. At least on the mRNA level, also no correlation between the corresponding 1249G > A polymorphism and MRP2 expression could be detected in normal kidney [88]. On the other hand, a possible interference of ritonavir, lopinavir and atazanavir with MRP4-/MRP2-mediated tenofovir efflux is not supported by certain *in vitro* data showing that none of these protease inhibitors aggravated tenofovir-induced cytotoxicity in hMRP2- or hMRP4-overexpressing MDCK cells, indicating a lack of interaction with these carriers. However, at least in the absence of serum, ritonavir did indeed significantly inhibit tenofovir efflux from hMRP4-HEK293T cells [89]. Based on their results, the latter authors attributed tenofovir nephrotoxicity in the context of highly active antiretroviral therapy (HAART) to drug-drug interactions at the level of hepatic transport and/or metabolism. Yet, given that tenofovir has been reported to be predominantly eliminated unchanged via the kidneys through filtration and secretion [90] and that impaired liver function was not found to alter tenofovir disposition [91], this assumption may not be correct, especially since ritonavir has indeed been shown to retard renal tenofovir elimination [92].

So far, data on interaction of the nucleoside phosphonate antivirals mentioned, as well as protease inhibitors, with human OATs other than OAT1 and OAT3, notably the apical OAT4, are lacking. This would be of particular interest as to better understand both the luminal efflux step not only for tenofovir, but also cidofovir (see above), as well as to identify potential targets for antiviral drug interactions.

### 3.3. Non-steroidal anti-inflammatory drugs

Nephrotoxicity induced by non-steroidal anti-inflammatory drugs (NSAIDs), many of which are known to interact with human OATs (recently reviewed in [6]), has frequently been reported [3,93]. However, in contrast to the early study by Muhalwas [94], renal adverse effects of NSAIDs have mostly been attributed to causes other than direct proximal tubular toxicity, such as hemodynamic changes due to inhibition of prostaglandin synthesis [93,95], allergic interstitial nephritis [96,97], or papillary necrosis [98]. Moreover, Rosenberger and co-workers have suggested that ischemia may contribute even to kidney injury primarily ascribed to direct tubulotoxicity [95]. Similarly, as pointed out by Silva, it is often difficult to establish whether tubular epithelial damage may be secondary to interstitial nephritis or is the primary event [99].

Although nephrotoxicity of NSAIDs is low when taken at therapeutic doses over short periods of time, NSAID-induced nephropathy presenting with papillary rather than proximal tubular damage has frequently been observed as a result of abusive NSAID consumption, or when renal perfusion was compromised [98,100]. Thus, NSAID-induced chronic and/or acute renal papillary necrosis has been reported for phenacetin, acetylsalicylate, fenoprofen, mefenamic acid, ibuprofen, and phenybutazone [98,100], several of which most potently interact with hOAT1 and/or hOAT3, while higher concentrations were found to also inhibit hOAT2 and hOAT4 (Table 1).

At least for the excessive use of phenacetin (or its active metabolite acetaminophen) in combination with aspirin, papillary necrosis has been established as the primary event [99]. Toxic free radicals resulting from acetaminophen metabolism are postulated to be the cytotoxic agents, and aggravation of the damage by salicylates appears to result from glutathione depletion [99,101], rather than drug-drug interactions at sites of renal transport. Interestingly, acetaminophen, acetylsalicylate and salicylate have all been found to be directly toxic to mouse inner-medullary collecting duct cells in culture, and cyclooxygenase inhibition alone could not account for this effect [102]. Since high doses of drugs are required to induce these effects, proximal tubular secretion followed by intratubular concentration may well contribute to the selective papillary damage. Although, to the best of our knowledge, actual transport of NSAIDs by members of the OAT family of carriers has never been demonstrated, potent inhibition of the basolateral exchangers OAT1 and OAT3, in particular, by a variety NSAIDs may implicate these transporters in their trans-tubular secretion [50,51](Table 1).

In this context it is interesting to note that phenacetin, a potent inhibitor of hOAT3, had been removed from the market by 1999, owing to its nephrotoxicity, whereas its metabolite acetaminophen, which only appears to interact rather weakly with hOAT1, has been reported not to be toxic to the kidney when taken at therapeutic doses [98]. 

Apical export of NSAIDs might be mediated by MRP2 and MRP4, which have been shown to be inhibited by a variety of NSAIDs, including ibuprofen and phenylbutazone [52,53,103](Table 1). Inhibitory potency was typically higher toward MRP4 than toward MRP2 [52]. That NSAIDs can indeed function as substrates of MRPs has for example been demonstrated by increased MRP2-ATPase activity in the presence of indomethacin [103]. OAT4, which is substantially more sensitive to inhibition by most NSAIDs tested than the basolateral OAT2 [50], represents an additional candidate efflux pathway into the tubular lumen.

### 3.4. Aristolochic acid

In 1992, two female patients were admitted to hospitals in Brussels (Belgium), with severe interstitial nephritis, resulting in terminal renal failure within a relatively short period of time. This rapidly progressing nephropathy could only be attributed to the slimming pills both women had been taking for a period of more than a year. Subsequently, it was suspected that in the medication containing herbs used in traditional Chinese medicine, *Stephania tetrandra* had probably inadvertently been replaced by *Aristolochia fangchi*, which contains aristolochic acid (AA)[104,105]. The nephrotoxic action of AA in humans had already been described in 1964 by Jackson and coworkers [106]. Despite the resulting ban of *Stephania tetrandra* from the Belgian market by the end of 1992, more than 100 cases of Chinese-herb nephropathy (CHN) were reported in Belgium in 1998 alone [107]. 

Several *in vivo* and *in vitro* experiments indicated that the main targets of AA are the proximal tubule cells. The histological hallmark of CHN detected in kidney biopsies of patients was proximal tubular atrophy. Lesions of this type of nephropathy were primarily found in the superficial cortex, the renal zone with a very high density of proximal tubules, subsequently progressing into deeper cortical regions [108]. Quantification of the activity of neutral endopeptidase (NEP), a marker enzyme of the proximal tubular brush border membrane, in urine of patients in earlier stages of the nephropathy, and in patients with severe renal failure (end-stage) due to AA administration revealed a 45% and 90% reduction of the NEP activity, respectively, compared to healthy control subjects. NEP is excreted in urine under physiological conditions and is taken as measure for the amount of renal enzyme [109]. Treatment of rabbits and rats with AA was found to result in flattening of the proximal tubular epithelium, as well as to severe tubular atrophy [110]. In rats, AA-induced tubular atrophy and development of interstitial fibrosis could be attributed to epithelial mesenchymal transformation (EMT). This was evidenced by a decrease in N- and E-cadherin expression, and a concomitant increase in the mesenchymal marker vimentin as well as the myofibroblast protein alpha-smooth muscle actin [111]. Consistent with the hypothesis that contamination with AA, known for its carcinogenic potential, was the underlying cause of CHN, both pure AA as well as the herbal mixture contained in the slimming medication induced tumors in rats [112]. Moreover, AA-specific DNA adducts could be detected in kidney samples of CHN patients [113]. AA-induced DNA adduct formation may be causally related to the morphological changes seen in renal tissue from CHN patients, as AA-treated opossum kidney (OK) cells were found to have reduced megalin surface expression and were positive for AA-related DNA adducts [114].

OK cells are not only a well established proximal tubule cell model for studies of the megalin-/cubilin-mediated endocytosis, but have also been documented to express critical components of the organic anion secretory pathway, thus exhibiting probenecid-sensitive intracellular accumulation as well as basolateral-to-apical transcellular flux of model organic anions such as ρ-aminohippurate [115]. As AA is an organic anion, its entry into the cells in the studies by Lebeau *et al.* might have been OAT-mediated [114]. Recently, Bakhiya *et al.* indeed demonstrated potent inhibition of hOAT1, hOAT3 and hOAT4 expressed in HEK293 cells by aristolochic acid I (AAI) with K_i_ values of 0.6, 0.5, and 20 µM, respectively [54]. Moreover, upon AA treatment, significantly higher levels of AA-specific DNA adducts were detected in all hOAT-overexpressing cells than in controls, an effect which could abolished by the presence of probenecid during the incubation with AA.

The preferential damage of proximal tubule cells in AA nephropathy may thus be due to hOAT1-/hOAT3-mediated cellular AA accumulation. Given its asymmetric operation, it remains to be determined whether hOAT4 can function as an apical efflux pathway for AA, or may rather aggravate cellular injury by mediating additional uptake from the tubular fluid. As yet, the role of hOAT2, as well as of MRP2 and MRP4 in the proximal tubular handling of AA and thus their potential impact on the progression of AA-induced nephropathy has not been resolved. 

## 4. Nephrolithiasis

Drug-induced nephrolithiasis in general appears to be a relatively rare event [116]. Nevertheless, a number of drugs known to interact with OAT-type transporters, including methotrexate, some antibiotics and certain antiviral therapeutics, have been more frequently associated with acute kidney injury due to intratubular crystal formation [2,4]. Risk factors include dehydration, metabolic changes affecting urinary pH, as well as, at least with respect to some compounds, calciuria (see below).

### 4.1. Methotrexate

Methotrexate (MTX) is used in the management of certain types of cancer as well as several auto-immune diseases, such as rheumatoid arthritis, inflammatory bowel disease or systemic lupus erythematodes [117,118,119,120]. For the treatment of malignancies in both adults and children, high-dose methotrexate is frequently used [121]. However, a number of sometimes fatal cases of MTX-induced acute renal failure have been reported, especially upon co-administration with NSAIDs, such as indomethacin and ketoprofen [122,123,124,125]. As stated by Widemann and Adamson in 2006, MTX-induced nephrotoxicity still occurs, albeit infrequently [121]. MTX-induced renal impairment appears to be primarily due to the precipitation of MTX and its metabolites in the tubular lumen, although direct tubular toxicity due to compromised apical MTX efflux has also been reported [4,121,126]. MTX is excreted mostly unchanged via the kidneys and renal elimination appears to be a function of both secretion as well as reabsorption [127,128]. As the solubility of MTX is low at acidic pH values, metabolic changes resulting in increased tubular acidification constitute a further risk factor in addition to high dosage. 

hOAT3 has been shown to be a high-affinity transporter for MTX uptake into renal proximal tubule cells [10,129], whereas hOAT1 was initially reported not to accept MTX as a substrate [13](Table 2). However, the failure to detect hOAT1-mediated MTX transport in the latter study might have been due to the low concentration (0.2 µM) used, since hOAT1 was later shown to have a low MTX affinity (K_m_ 554 µM/724 µM [51,129]). Thus, hOAT1 may well contribute significantly to basolateral MTX uptake into renal proximal tubule under high-dose methotrexate administration as used in cancer therapy, resulting in mean peak MTX plasma concentrations of 1mM and above have been determined [130]. As yet, it is unclear if any back-leak of MTX into the circulation via hOAT2 can be completely excluded, as the inability of this carrier to mediate MTX flux has so far only been indicated in one report, referring to unpublished data with no indication of the concentration applied [51].

**Table 2:** Interaction of drugs associated with nephrolithiasis with human renal OATs and other proximal tubular transporters. All values are in µM. T: transport demonstrated; n.t.: not transported; I: inhibition shown; n.i.: no inhibition; *: K_m_; ^●^: K_i_; ^◊^: IC_50_; *^a^*: referral to unpublished data in [51].

Substrate	OAT1	OAT2	OAT3	OAT4	Other Transporters			References
					MRP2	MRP4	Additional Carriers	
**Cytostatics**								
methotrexate (MTX)	*724; *554; n.t.	*^a^*n.t.; T	*17.2; *10.9; *21.1	*17.8	*2500–3000; *250; *480	*220; *220; *1300	hOATP4C1: T	**[10,13,19,51,52,103,129,131,132,133,134]**
**ß-Lactam Antibiotics**								
Ceftriaxone	^●^230	^●^6760	^●^4390	^●^2380	T		PEPT1: n.i.	**[29,32,33,36]**
**Carboxyfluoroquinolones**								
Ciprofloxacin	n.i.		I				MATE1: n.t.; ^◊^231; MATE2-K: n.t.; ^◊^98.7	**[135,136]**
**Antivirals**								
Acyclovir	*342	n.i.	n.t.; I	n.i.			MATE1: *2640; MATE2-K: *4320	**[136,137]**

Reduced-folate carrier 1 (RFC1), known to be expressed basolaterally in mouse renal tubules [138], likely complements OAT3 (and OAT1) in contraluminal MTX uptake also in the human kidney, as hRFC expression induces MTX uptake and confers MTX sensitivity to MTX-resistant cells [139,140]. In rat renal cortical slices, RFC and Oat3 contributed about equally (approx. 30% and 30–50%, respectively) to MTX-uptake at a low concentration (500 nM), with Oat1 playing only a minor role [141]. As a further basolateral MTX transporter potentially contributing to the uptake step in renal MTX secretion, hOATP4C1 has been identified [134].

Luminal MTX efflux may in part be mediated by OAT4 [51], unless precluded by the carrier's asymmetry, in conjunction with MRP2 and MRP4, although the latter exhibit MTX affinities about 2–3 orders of magnitude lower than determined for OAT4, with MRP2 typically displaying somewhat lower K_m_ values than MRP4 (Table 2). Taken together, at low doses of MTX, the SLC22 family members hOAT3 and hOAT4 are likely involved in its transepithelial secretion. Under high-dose MTX therapy, hOAT1 may also play a significant role and could thus contribute to MTX crystalluria and tubular damage.

As for the interaction between MTX and NSAIDs, increased systemic toxicity seems evident from common renal secretory mechanisms. In contrast, increased toxicity of MTX to the kidney under this condition is less easily explained. However, although hOAT3, as well as hOAT1, are highly sensitive to inhibition by most NSAIDs tested [50](cf. Table 1), Takeda *et al.* have indicated that due to the high plasma protein binding of NSAIDs, *in vivo* only indomethacin, phenylbutazone and salicylate, but not e.g., ibuprofen or ketoprofen, may interfere with hOAT3-dependent renal MTX secretion [51]. On the other hand, since the K_i_ values of NSAIDs for inhibition of the reduced folate carrier, at least in the rat, are about one to two orders of magnitude higher (70–310 µM) than those for Oat3 and Oat1 [141], interference of NSAIDs with proximal tubular secretion of MTX is probably even less extensive than assumed based on consideration of OAT3 (and OAT1) alone [28]. Thus, it would be important to elucidate the relative contribution of RFC and OAT3/OAT1 to cellular MTX uptake and thereby transepithelial secretion in human renal proximal tubules at clinically relevant concentrations attained under both low-dose and high-dose MTX therapy. Notably, not only OAT1, 3 and 4, but also MRP2 and MRP4 have been shown to be potently inhibited by a variety of NSAIDs, including indomethacin (IC_50_ values of 0.06 µM for MRP2 and 6 µM for MRP4) and ketoprofen (IC_50_ values of 1.4 and 470 µM for MRP2 and 11.9 µM for MRP4)[52]. 

However, as mentioned above, pharmacokinetic evidence indicates that MTX is transported birectionally in the human renal tubules. At least part of the reabsorptive flux of reduced folates, such as 5-methyltetrahydrofolate and MTX, appears to occur in the proximal tubule, as indicated by studies on apical-to-basolateral flux of 5-methyltetrahydrofolate in cultured human proximal tubule cells [142,143], and likely involves the proton-coupled reduced folate carrier, PCFT. hPCFT has been shown to be expressed in renal proximal tubule cells [144] and, when transfected into MDCK cells, is targeted to the apical membrane, while hRFC, consistent with its localization in mouse kidney, is targeted basolaterally [145]. PCFT has been shown to accept methotrexate as a substrate, even though its MTX transport activity appears to be low, and the affinity [146], which is highly pH-dependent, is not known for the mildly acidic conditions normally encountered in the proximal tubule. Moreover, to our knowledge it is still unknown whether NSAIDs can interfere with hPCFT-mediated reabsorption of MTX from the tubular fluid and thereby enhance kidney injury.

Interestingly, however, another luminal transporter further along the renal tubule, namely OATP1A2, has recently been shown to transport MTX and implicated in its reabsorption [147]. OATP1A2 has been immunolocalized to the apical membrane of renal distal tubules [148], and its MTX affinity (K_m_ 457 µM) is consistent with therapeutically induced intratubular MTX concentrations in this nephron segment expected to be up to 100 times higher than in plasma [147]. Although so far the sensitivity of OATP1A2 to NSAID inhibition has also not been reported, Oatp1a1 (also known as oatp1) as one of its rat homologs [149] has shown to be significantly inhibited by indomethacin concentrations in the micromolar range [150]. If human OATP1A2 were similarly sensitive, it appears possible that even low rates of OAT1-/OAT3-mediated proximal tubular secretion of indomethacin might suffice to achieve inhibitory levels in the distal nephron. In summary, similar to increases in plasma MTX concentrations induced by NSAIDs, their potentiating effect on nephrotoxicity is still incompletely resolved and requires consideration of both proximal tubular secretory mechanisms as well as potential reabsorptive pathways also in later parts of the nephron.

### 4.2. Crystal-nephropathy caused by ciprofloxacin and ceftriaxone

Ciprofloxacin crystalluria had orginally been considered an unlikely event in humans, as precipitation was only observed at alkaline pH values >7.3 [151]. Nevertheless, a number of cases of acute renal failure have been attributed to ciprofloxacin nephrolithiasis, including several more recent ones [152,153,154,155]. Ciprofloxacin is a widely used broad-spectrum fluoroquinolone antibiotic, which is among the three drugs recommended by the WHO for the treatment of dysentery in children, a prime cause of morbidity especially in developing countries [156]. Ciprofloxacin is preferentially administered orally and renal elimination accounts for ~50% under this condition, about two-thirds of which are the parent compound [157]. Following intravenous injection, ~60% are eliminated unchanged via the kidneys. Mean serum protein binding has been determined to amount to about 40% [157] and is given as 20–40% by the Bayer package insert 2009, indicating that a substantial fraction of ciprofloxacin is likely filtered at the glomerulus. Yet, renal clearance assayed only upon intravenous administration greatly exceeded the creatinine clearance, depending on dosage by up to 200%, indicating substantial net tubular secretion [157], which is consistent with the observation that co-administration of the organic anion transport inhibitor probenecid significantly decreases renal ciprofloxacin excretion by ~60% [158].

One likely candidate to mediate basolateral ciprofloxacin uptake in the renal proximal tubule is hOAT3, which has recently been shown to be sensitive to ciprofloxacin inhibition (~60% inhibition at 10,000-fold excess over the substrate), whereas hOAT1-mediated transport was not significantly affected [135]. Additional support for an involvement of Oat3, but not Oat1, was provided by the demonstration that expression of mouse Oat3 in CHO cells resulted in significantly (5-fold) higher ciprofloxacin uptake compared to controls, which was abolished in the presence of probenecid, whereas mouse Oat1 did not. Moreover, following injection of a single bolus of ciprofloxacin, significantly increased ciprofloxacin plasma concentrations were detected in Oat3 knockout relative to wildtype mice [135].

Based on one study on the ciprofloxacin resistence in mouse macrophages, MRP4 might be more likely than MRP2 to play a role in apical ciprofloxacin extrusion in the renal proximal tubule. It will be important to verify that human OAT3 and MRP4 do indeed behave similarly as their mouse counterparts with respect to ciprofloxacin handling, which may not necessarily be the case. In this context it is noteworthy that, while the rat cation transporter MATE1, expressed in the apical membrane of proximal tubule cells [159], has been found to be able to transport ciprofloxacin, this does not seem to be apply for the human MATE1 or MATE2-K, although they were potently inhibited by it [136,160]. Interaction of ciprofloxacin, which is zwitterionic at physiological pH [161], with at least some organic cation transporters also indicates, that others, like e.g., hOCT2 may also not be completely disregarded as potential additional renal ciprofloxacin transporters.

There are several reports in the literature associating the third-generation cephalosporin ceftriaxone with nephrolithiasis—generally believed to be due to the formation of insoluble precipitates with calcium—especially in children, but impairment of renal function was usually not observed [162,163,164]. In isolated cases, however, ceftriaxone crystalluria has been fatal in neonates and infants, leading the US FDA in 2007 to recommend not using calcium-containing infusions simultaneously with ceftriaxone [165]. Close monitoring for kidney stone formation during ceftriaxone therapy has also been suggested [162]. Ceftriaxone is widely used to treat childhood infections, and is another WHO-recommended drug to treat dysenteria [156]. Although like other cephalosporins, ceftriaxone is eliminated to a significant extent (approx. 40%) via the kidneys [166,167], it differs in a substantially longer elimination half-life than reported for most other cephalosporins [166]. This may in part be due to its high serum protein binding, limiting glomerular filtration [166,167]. Nevertheless, glomerular filtration still accounts for about 70% of the total renal ceftriaxone clearance, while the remainder is mediated by probenecid-sensitive tubular secretion [167]. Important players in the proximal tubular handling of cephalosporin antibiotics in general have already been discussed above in the context of direct tubular toxicity.

As mentioned, human OAT3 expressed in HEK293 cells mediated uptake of all cephalosporins tested, albeit the extent substantially differed between compounds when assayed at equal concentrations [34]. However, while ceftriaxone was not included in this study, it exhibited a far lower inhibitory potency than other cephalosporins on hOAT3-mediated transport (K_i_ 4.39 mM) in another [32]. It thus appears unlikely that hOAT3 plays a significant role in ceftriaxone secretion at clinically relevant plasma concentrations [166,168]. hOAT1, on the other hand, appeared to be able to accept only a limited number of cephalosporins as substrates, which—additionally—were transported rather poorly, with uptake never higher than twice the control level in hOAT1-expressing HEK293 cells [34]. Thus, despite a similarly low K_i_ value determined for ceftriaxone interaction with hOAT1 as for cefazolin (230 µM and 180 µM, respectively)[32], hOAT1-mediated ceftriaxone transport may still be low or absent, as cefazolin is also not an hOAT1 substrate. hOAT2 is unlikely to play a role in renal ceftriaxone handling *in vivo* as well, given the high reported K_i_ value of over 6 mM [32]. Taken together, these data are in line with the long elimination half-life of ceftriaxone, and indicate that OAT-dependent basolateral ceftriaxone uptake as the first step in transtubular secretion is not an important mediator in ceftriaxone nephrotoxicity. It may therefore rather depend on—albeit limited—glomerular filtration, with the possible contribution of as yet unidentified secretory pathways, involving MRP2 [29], and maybe MRP4, at the luminal side.

### 4.3. Acyclovir-induced nephrolithiasis

Crystal nephropathy is a well-known adverse side-effect of the antiviral drug acyclovir with its low solubility in urine, in particular when applied intravenously at high doses [4,66]. Acyclovir is a guanosine analogue widely used in the treatment of infections with members of the herpesvirus family. Binding of acyclovir to plasma proteins has been determined to be low, and elimination occurs to an average of ~65% as unchanged drug via the kidneys by glomerular filtration as well as tubular secretion [169]. The importance of tubular transport in renal acyclovir excretion is supported by its substantial inhibition when co-administered with probenecid or cimetidine [170,171]. Acyclovir has been shown to be a substrate of human OAT1, albeit with an affinity about one order of magnitude lower than the nucleotide analogues [137], possibly in part due to its lack of a negative charge. In contrast, neither hOAT3, nor hOAT2 or hOAT4 were able to transport acyclovir, although hOAT3 was sensitive to acyclovir inhibition [10,137]. Interestingly, acyclovir was also found to be transported by hOCT1, which is, however, only expressed to very low levels in human kidney [8,172], whereas hOCT2, which is strongly expressed in kidney [8,172,173], did not mediate acyclovir uptake [137]. Regarding apical efflux, there is no information regarding MRP2- or MRP4-mediated acyclovir transport. However, MRP4 overexpression has been shown to confer resistance to another guanosine analogue, ganciclovir [174], making MRP4 a candidate export mechanism for acyclovir into the tubular lumen. Although the human organic cation/proton exchangers MATE1 and MATE2-K have both been reported to accept acyclovir as a substrate, they do so with rather low affinity (K_m_ values of 2.64 and 4.32 mM, respectively)[136]. Hence, it is questionable whether they could indeed significantly contribute to proximal tubular acyclovir secretion at the cellular concentrations that will be attained at clinically relevant plasma levels in the low micromolar range [169,171,175]. 

## 5. Concluding Remarks

OAT family members clearly play a critical role in the renal transport of a variety of drugs with well-known nephrotoxic potential. Certainly, their importance might be overestimated in isolated cases where major alternative transport pathways may not have been identified, as exemplified by renal MTX handling. However, not recognizing a possible interaction of clinically relevant drugs with OATs, or any other transporter involved in renal xenobiotic handling, is potentially far more consequential. Thus, toxicity of compounds accumulated in renal proximal tubule cells by OAT1- and/or OAT3-mediated transport may only become apparent when (apical) extrusion is impaired. This could be due to polymorphisms affecting expression and/or function of the mechanism/s responsible, or their unanticipated inhibition by co-administered agents. It thus seems imperative for all newly developed drugs to be tested against any potentially interacting transport mechanism available for functional analysis. Clearly, transport affinity, turnover rate and sensitivity to inhibition in heterologous expression systems will never accurately reflect the *in vivo* situation, even if assayed under conditions resembling human serum. Yet, together with knowledge of the relative expression levels of different transporters and driving forces in the tissue, such tests may still give a good indication of, e.g., levels of cellular drug accumulation or drug interactions to be expected. Moreover, they may provide important clues on the parameters to be analysed in patients, when certain drugs are to be given, leading to more personalized and hopefully less toxic drug therapies. 
